# Changing patterns of mortality during the COVID-19 pandemic: Population-based modelling to understand palliative care implications

**DOI:** 10.1177/0269216320944810

**Published:** 2020-07-24

**Authors:** Anna E Bone, Anne M Finucane, Javiera Leniz, Irene J Higginson, Katherine E Sleeman

**Affiliations:** 1 Cicely Saunders Institute of Palliative Care, Policy and Rehabilitation, King’s College London, London, UK; 2 Marie Curie Hospice, Edinburgh, UK; 3 Usher Institute, Old Medical School, The University of Edinburgh, Edinburgh, UK

**Keywords:** Palliative medicine, pandemics, mortality, population health, nursing homes, health services

## Abstract

**Background:**

COVID-19 has directly and indirectly caused high mortality worldwide.

**Aim:**

To explore patterns of mortality during the COVID-19 pandemic and implications for palliative care, service planning and research.

**Design:**

Descriptive analysis and population-based modelling of routine data.

**Participants and setting:**

All deaths registered in England and Wales between 7 March and 15 May 2020. We described the following mortality categories by age, gender and place of death: (1) baseline deaths (deaths that would typically occur in a given period); (2) COVID-19 deaths and (3) additional deaths not directly attributed to COVID-19. We estimated the proportion of people who died from COVID-19 who might have been in their last year of life in the absence of the pandemic using simple modelling with explicit assumptions.

**Results:**

During the first 10 weeks of the pandemic, there were 101,614 baseline deaths, 41,105 COVID-19 deaths and 14,520 additional deaths. Deaths in care homes increased by 220%, while home and hospital deaths increased by 77% and 90%, respectively. Hospice deaths fell by 20%. Additional deaths were among older people (86% aged ⩾ 75 years), and most occurred in care homes (56%) and at home (43%). We estimate that 22% (13%–31%) of COVID-19 deaths occurred among people who might have been in their last year of life in the absence of the pandemic.

**Conclusion:**

The COVID-19 pandemic has led to a surge in palliative care needs. Health and social care systems must ensure availability of palliative care to support people with severe COVID-19, particularly in care homes.

**What is already known about the topic?**

- The COVID-19 pandemic has directly and indirectly resulted in high mortality in many affected nations.
- Internationally, the response has been focused on prevention and curative treatments, with little emphasis on palliative care needs of people dying during the COVID-19 pandemic.
- We do not know how many of those dying with COVID-19 would have been in their last year of life in the absence of the pandemic, and this group may have distinct care needs.

**What this paper adds?**

- The number of people dying in care homes increased by 220% during the first 10 weeks of the COVID-19 pandemic in England and Wales; many of these deaths were ‘additional deaths’, which is associated with the COVID-19 pandemic but not directly reported as a result of COVID-19.
- We estimate that just over one in five of all COVID-19 deaths occurred among people who might have been in their last year of life in the absence of the pandemic.

**Implications for practice, theory or policy**

- Health and social care systems must ensure availability of palliative care to support people with severe COVID-19, particularly in care home settings.
- The need for integrated models of palliative care in care home settings is key, and research to underpin these models is needed.

## Introduction

Approaches to death and dying reveal much of the attitude of society as a whole to the individuals who compose it (Cicely Saunders, 1984).^1^ The COVID-19 pandemic has led to an unprecedented surge in mortality around the world, with some countries such as United Kingdom, the United States and Italy particularly affected. In the United Kingdom and elsewhere, the government response has been focused on prevention and curative treatments. There has been very little emphasis internationally on care needs, including palliative care needs, for people dying during the COVID-19 pandemic.^2^

Clinical research and observation has found that people dying with COVID-19 are symptomatic, frequently experiencing breathlessness and agitation near the end of life.^3^ Clinical uncertainty around the illness trajectories of those infected increases the complexity of clinical decision-making and communication with patients and family.^4^ Deaths occurring during the COVID-19 pandemic are likely to be associated with poorer bereavement outcomes for family and friends and higher distress and support needs among frontline health and social care staff.^5^ There is an important role for palliative care, but the palliative care needs of the population have not been described.

Large-scale population-based studies exploring patterns of mortality are important for anticipating numbers of people in need of care and for informing governments about the need to plan and reorganise services.^6,7^ Previous research suggests that up to 82% of people dying in a typical year have palliative care needs.^8^ However, the emergence of COVID-19 has changed patterns of mortality. We currently lack an understanding of the palliative care needs for people dying during the COVID-19 pandemic at a population level.

In England and Wales, official mortality data from the Office for National Statistics (ONS) reveal that during the first 10 weeks of the pandemic (7 March to 15 May 2020), there were over 41,000 COVID-19 deaths, most occurring in hospitals (65%), with 28% in care homes and few elsewhere (7%).^9^ The ONS estimated that there were also over 14,000 ‘additional’ deaths, which were deaths over and above the number of deaths expected based on the 5-year average and which were not certified as caused by COVID-19.^9^ Most people dying with COVID-19 are older with underlying health conditions. The extent to which people dying with COVID-19 may have been in their last year of life in the absence of the COVID-19 pandemic has stimulated considerable debate.^10^ Estimating this could help inform resource allocation and service planning, since the goals of care for those with a pre-existing advanced progressive or terminal illness may differ from those with no serious health concerns prior to a COVID-19 infection.

This study aims to explore patterns of mortality during the first 10 weeks of the COVID-19 pandemic in England and Wales (7 March to 15 May 2020) to understand implications for palliative care, service planning and research. The objectives are (1) to explore trends in place of death; (2) to explore the age and gender distribution of baseline deaths, COVID-19 deaths and additional deaths; (3) to estimate the proportion of people who died from COVID-19 who would have been in their last year of life, and differences by age; and (4) to use this information to discuss implications for palliative care provision, service planning and research.

## Methods

### Design and setting

Descriptive analysis and population-based modelling using routinely collected mortality data for England and Wales.

### Data

We used publicly available data from the ONS including the following:

- Weekly counts of registered deaths in England and Wales by age group from 2015 to 2019.
- Weekly provisional counts of registered deaths in England and Wales in the weeks corresponding to the COVID-19 pandemic, between 7 March (week 11) and 15 May 2020 (week 20), according to the date of registration.^9^ The week number refers to the week within the calendar year, for example, week 1 is the first week of January, which allows for comparison between the same period in previous years. We included data from the week beginning March 7, as this was the first week that deaths from COVID-19 were registered in England and Wales, until the latest available data (week 20). ONS death registration figures are published 11 days after the week ends due to the processing of data.^9^ Available data include all deaths and COVID-19 deaths by age, gender and place of occurrence over this time period.
- Principal population projections of the total population of England and Wales by age for 2020 (2018-based).^11^

Death registration data have been used in previous studies examining national trends in place of death.^6^ In response to the pandemic, the ONS has released weekly death registration data, including deaths involving COVID-19 and place of occurrence for all deaths. Guidance for professionals completing death certificates states that COVID-19 should be included as a contributing or underlying cause of death, when this is suspected even in the absence of laboratory confirmation.^12^

### Analysis

We first defined the following mortality categories: baseline deaths, COVID-19 deaths and additional deaths, as detailed in Box 1.

**Box 1.** Definitions of mortality categories.

Terms	Definition
Baseline deaths	Deaths that would be expected to occur in a typical year without a pandemic. For this analysis we used the average number of deaths that occurred in the same period over the past 5 years.
COVID-19 deaths (a) COVID-19 deaths among annual baseline deaths	Deaths officially registered as with or from COVID-19. This category includes a subgroup: (a) *People who died from COVID-19 who might have been in their last year of life in the absence of the pandemic.*
Additional deaths	Deaths in excess of baseline deaths that are not accounted for by deaths certified as being as a direct result of COVID-19. This group might include deaths due to COVID-19 but not recorded as such, or deaths indirectly related to the pandemic, for example, through avoidance of hospital care.

The numbers of people in each mortality category were calculated for England and Wales between 7 March (week 11) and 15 May 2020 (week 20) as follows:

#### 1. Baseline deaths

We estimated the baseline deaths according to the average deaths in the 10-week period of interest (weeks 11 to 20) over the years 2015 to 2019, by age and gender.

#### 2. COVID-19 deaths

Data on COVID-19 deaths were obtained from the ONS weekly registered deaths, which include deaths by age, gender and place of occurrence.^9^ The ONS produces figures for deaths involving COVID-19 based on any mention of COVID-19 on the death certificate, including when the physician suspects COVID-19 based on symptoms but where no test was available.^9^ These figures include deaths outside of hospital.

##### a) People who died from COVID-19 who might have been in their last year of life in the absence of the pandemic

First, we derived an estimate of the proportion of the population infected in each age group by re-arranging the following equation:

*COVID-19 deaths = total population x proportion infected x infection fatality ratio*.

The information we used in this equation included the number of registered COVID-19 deaths reported by ONS;^9^ the total population from the ONS principal projections^11^ and infection fatality ratios by 10-year age groupings obtained from the work by Ferguson et al.^13,14^ (supplementary Table 1).

Second, we hypothesised that someone who is in their last year of life will have an increased risk of dying from COVID-19 compared to someone in the same age group who is not in their last year of life. To account for this, we applied a risk of dying multiplier to this group based on evidence of increased risk of death among people with serious conditions (supplementary Table 2). We applied a range of multipliers, with our primary estimate based on the median risk of death across different conditions.^15^ Therefore, the number of people who die with COVID-19 who would be expected to die within a year was calculated using the following equation:

*COVID-19 deaths among those in their last year of life = baseline deaths x proportion infected x infection fatality ratio x risk of dying multiplier*

#### 3. Additional deaths

We calculated additional deaths by subtracting the estimated baseline deaths and COVID-19 deaths from all registered deaths on a per week basis over the 10-week period of interest (weeks 11–20). This was calculated for each age and gender group separately to identify additional deaths within different demographic categories. Where this resulted in a negative value, the estimated baseline figure was reduced by this amount and the value for additional deaths was zero.

##### Age, gender and place of death

To understand the age and gender distribution of the COVID-19 and additional deaths, we grouped data into four age groups (<45, 45–64, 65–74 and ⩾ 85 years) and compared them with baseline deaths over the period of interest (weeks 11–20). We described trends in place of occurrence of deaths using the ONS defined categories of ‘hospital’ (acute and community, not psychiatric), ‘care home’ (nursing and residential), ‘home’, ‘hospice’ and combined ‘other communal’ and ‘elsewhere’. To estimate additional deaths by place of death over the period, we used place of death data for week 11 to estimate baseline place of death, as weekly place of death information was unavailable for previous years.

### Patient and public involvement

We sought the views and experiences of members of our patient and public network regarding their concerns around COVID-19 pandemic. These consultations informed the focus and design of this study. Patient and public involvement (PPI) representatives provided feedback on the article.

### Ethics

This study uses routinely collected anonymised and publicly available data. No ethical approvals were necessary.

## Results

**Figure 1.**
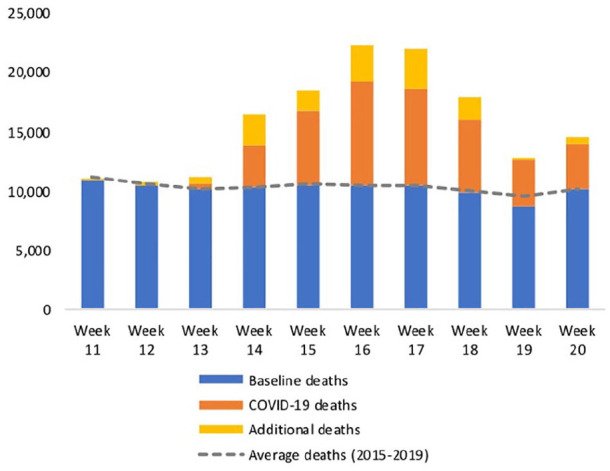
All registered deaths during the COVID-19 pandemic between 7 March and 15 May 2020 (weeks 11–20) in England and Wales.

Between 7 March and 15 May 2020 (weeks 11–20) in England and Wales, there were 157,239 deaths registered, of which 41,105 were from COVID-19. We estimate that this included 101,614 baseline deaths (as estimated by average weekly deaths over the same period during 2015–2019) and 14,520 additional deaths (Figure 1). The lower number of deaths observed in week 19 is likely due to a public holiday in that week which delayed the registration of deaths.^9^

### Place of death during the pandemic

**Figure 2.**
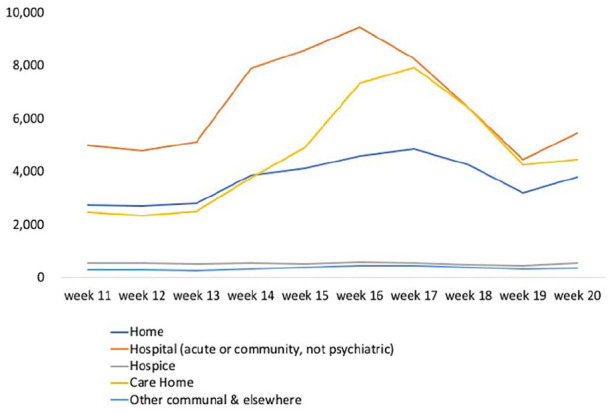
All registered deaths during the COVID-19 pandemic between 7 March and 15 May 2020 (weeks 11–20) in England and Wales by place of occurrence.

Where people die in England and Wales has changed (Figure 2). Taking all deaths together, weekly hospital deaths increased by 90% from week 11 (n = 4975) to a peak in week 16 (n = 9434). We observe a 77% increase in home deaths between weeks 11 (n = 2725) and 17 (n = 4834), while care home deaths increased by 220% during the same period (week 11 n = 2471 and week 17 n = 7911). In week 18, care homes temporarily became the most common place to die. Hospice deaths fell by 20% between week 11 (n = 553) and week 19 (n = 441), representing 3% of deaths during the 10-week period.

**Figure 3.**
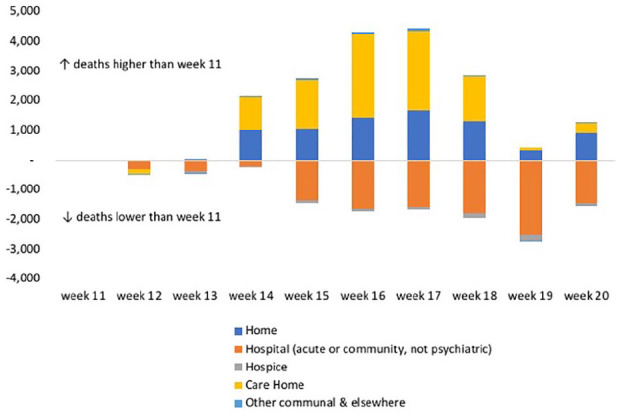
Difference in the number of additional deaths (not attributed to COVID-19) in weeks 12–20 compared to week 11 in 2020, by place of occurrence.

Most COVID-19 deaths occurred in hospitals (65%) and care homes (28%), with few occurring at home and hospice (5% and 1%, respectively). The location of the additional deaths has not been described. Comparing place of death for additional deaths occurring in weeks 12–20 with week 11, we found the majority of additional deaths occurred in care homes (56%) and at home (43%) (Figure 3). During weeks 12–20, there were fewer additional deaths in hospitals and hospices compared to week 11, indicating that there were fewer non-COVID-19 deaths in these settings than usual.

### Mortality categories by age and gender

**Figure 4.**
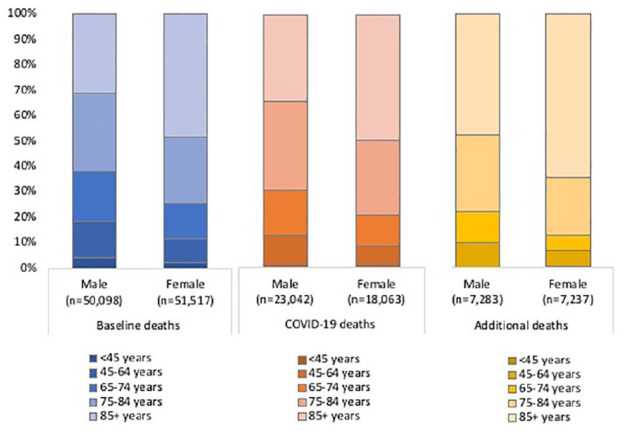
Age and gender distribution of baseline deaths, COVID-19 deaths and additional deaths between 7 March and 15 May 2020 (weeks 11–20) in England and Wales.

We found the age distribution of COVID-19 deaths to be overall older compared to baseline deaths, with 74% of COVID-19 deaths aged ⩾ 75 years compared to 68% of baseline deaths (Figure 4). Additional deaths are older still, with 83% aged ⩾ 75 years and 56% aged ⩾ 85 years. A greater proportion of COVID-19 deaths are male (56%), compared to 49% of baseline deaths and 50% of additional deaths.

### People who died from COVID-19 who might have been in their last year of life in the absence of the pandemic

Among the people who are registered as dying with COVID-19, we have estimated a group who would be in their last year of life in the absence of the pandemic. Using our primary (and lower and upper) estimates for the risk of dying multiplier, we found that 22% (13%–31%) of people who died with COVID-19 over the period of interest were likely to be in their last year of life (Table 1 and supplementary Figure 1). This increases with age, such that by ≥ 80 years, 33% (19%–47%) of people who die with COVID are likely in their last year of life in the absence of the pandemic.

**Table 1.** People who died from COVID-19 over the 10-week period from 7 March to 15 May 2020 who might have been in their last year of life in the absence of the pandemic.

Age	Total population of England and Wales^11^	Baseline deaths over 12 months (based on the 5-year average)	Total COVID-19 deaths up to 15 May	Infection fatality ratio (based on the work by Ferguson et al 2020)^13^	Estimated proportion infected with COVID-19 over the period	COVID-19 deaths among annual baseline deaths n (% of all COVID-19 deaths)		
						Multiplier of 3.5 – primary estimate	Multiplier of 2 – lower estimate	Multiplier of 5 – upper estimate
0–9	7,142,647	3305	3	0.00%	2.1%	0 (0%)	0 (0%)	0 (0%)
10–19	6,908,476	2823	10	0.01%	2.4%	0 (0%)	0 (0%)	0 (0%)
20–29	7,654,985	4955	64	0.03%	2.8%	0 (0%)	0 (0%)	0 (0%)
30–39	7,973,235	4955	181	0.08%	2.8%	0 (0%)	0 (0%)	1(0%)
40–49	7,495,052	18,176	591	0.15%	4.3%	5 (1%)	3 (0%)	7 (1%)
50–59	8,080,681	31,396	1953	0.6%	4.0%	27 (1%)	15 (1%)	38 (2%)
60–69	6,371,654	59,543	4150	2.2%	3.0%	136 (3%)	78 (2%)	194 (5%)
70–79	5,149,885	119,014	9431	5.1%	3.6%	763 (8%)	436 (5%)	1090 (12%)
80 +	3,052,619	286,883	24,722	9.3%	8.7%	8132 (33%)	4647 (19%)	11,617 (47%)
Total	59,829,234	531,050	41,105	–	–	9062 (22%)	5179 (13%)	12,946 (31%)

## Discussion

### Main findings

Using routine data and modelling scenarios to understand mortality patterns during the COVID-19 pandemic, we highlight that care homes temporarily became the most common place to die in England and Wales, and that hospital and home deaths increased by over 50%, while deaths in hospices fell by 20%. We show that people in the additional deaths category are proportionately more likely to be older, and to die in care homes and at home, than those who are certified as dying with COVID-19. Finally, we estimate for the first time that just over one in five COVID-19 deaths occur among people who might be in their last year of life in the absence of the pandemic, and that this increases to a third of those aged over 85 years.

### Implications for palliative care

Deaths in hospitals and care homes and at home increased during the first weeks of the COVID-19 pandemic. Rapid adaptation of models of palliative care is needed at the first sign of subsequent waves of the COVID-19 pandemic or future pandemics to support the increased number of people dying in these settings. Guidance on remote consultations in primary care^16^ and on managing distressing symptoms in the community^17^ is available to help support care for people with suspected COVID-19, but challenges remain in terms of workforce and equipment. A pilot study of palliative care delivered in care homes via teleconference supported the feasibility of this approach where required.^18^ Specialist palliative care provision in care homes is currently variable,^19–21^ and guidance to support palliative care for people with COVID-19 in nursing homes has been found to be lacking and limited in focus.^22^ In contrast, deaths in hospices fell during the time period. A similar phenomenon was found during the 2003 SARS pandemic and highlighted the need for seamless continuity of care between settings.^23^ Systems that enable hospice resources to rapidly shift from inpatient to community settings are likely to be important in future pandemic peaks.^24^

Little has been described about the characteristics of the additional deaths that have occurred during the COVID-19 pandemic. The aetiology of these deaths is not known, in particular whether they are undiagnosed COVID-19 deaths or deaths associated with the pandemic without being a result of the disease (e.g. due to avoidance of urgent healthcare).^25^ There is emerging evidence that older people with COVID-19 may present with non-specific and atypical symptoms such as delirium or low-grade fever.^26^ We found that the additional deaths group comprises predominantly older people in care homes, suggesting that these may be undiagnosed COVID-19 deaths. Irrespective of aetiology, this group are likely to have significant palliative care needs. The characteristics of the additional deaths population is likely to change as the pandemic progresses, and deferred harm from missed routine check-ups or screening becomes more likely.^27^

At a population level, we estimate that 22% of COVID-19 deaths occur among people who might have been in their last year of life in the absence of the pandemic, and that among those aged > 85 years, 33% might be in their last year of life. For those with advanced conditions living at home and in care homes, guidelines recommend that general practitioners (GPs), community healthcare staff and community geriatricians urgently review advance care plans with patients and their families and discuss their care preferences in the case of contracting COVID-19.^17,28^ These discussions are challenging, particularly when conducted over the telephone. Specialist palliative care services may play a role in offering advice and support for conducting difficult conversations in difficult situations.

While the focus of this article is COVID-19, it should be noted that baseline deaths outnumber COVID-19 and additional deaths. Previous modelling has shown that around 69%–82% of the baseline group are likely to have palliative care needs.^8^ COVID-19 will impact this group, for example, through fewer face-to-face contacts and potentially less availability of specialist palliative care support, as services are suddenly stretched beyond their usual bounds. Informal caregivers are likely to play an important role in caring for those in home settings and may need additional support.^29^

Across all mortality categories, there is the need for bereavement support as a result of higher prevalence of complicated grief.^30^ There will be the typical grief associated with death and dying, but on a much larger scale. In addition, there will also be novel grief processes, linked to social isolation and distancing, self-blame around infection and not being able to attend funerals.^31^ Palliative care specialists with particular expertise in bereavement support have a role in supporting families in coping with bereavement and supporting colleagues to deliver this care alongside other relevant organisations.

Internationally, the COVID-19 pandemic has affected countries at different times and to varying degrees. The United Kingdom has experienced particularly high mortality during the COVID-19 pandemic.^32^ A common feature across nations affected by COVID-19 is the high proportion of COVID-19 deaths among care home residents, for example, 34% in South Korea and 51% in France.^33^ All countries severely affected by COVID-19 will have a surge in need for palliative care, though country-level factors, such as socio-demographic characteristics, will influence the magnitude of this. National guidance on providing palliative care during pandemics, taking into account the structure of the health and social care system and level of integration of palliative care, is essential.^34^

### Strengths and limitations

This is the first article to estimate and characterise population-level patterns of mortality during the COVID-19 pandemic in relation to palliative care. We have produced simple models with explicit assumptions to raise questions and stimulate discussion. We calculated additional deaths for each age and gender group separately to improve precision. However, this study has limitations. We have been limited by the available official data, including the lack of historical data on place of death by week in England and Wales. Death registration figures can be affected by external factors such as public holidays, delays in processing or delays due to investigations by the coroner. Therefore, the death figures included in this analysis may underestimate the true number of deaths over this period. We have used data for England and Wales, and patterns are likely to vary by country and region, particularly with respect to care home deaths. The infection fatality ratio and risk of dying multipliers for those in their last year of life were based on the latest available data, and this might change over the course of the pandemic. Most of the underlying evidence for the multiplier is based on hospital studies; those dying with COVID-19 in care homes and at home are underrepresented.^15^

This study focused on mortality only. Other aspects of palliative care are important to be considered, for example, symptom control, support with difficult conversations, advance care planning, and complex clinical decision-making.^35^ There are also longer-term needs among those surviving COVID-19, such as rehabilitation and palliative care, which are likely to be significant and important for service planning but were not modelled here. We have produced population-based models that are useful for strategic health service planning but are not directly applicable to clinical decision-making at an individual level. For example, while we have estimated the number of people dying from COVID-19 who might have been in their last year of life in the absence of the pandemic at the population level, we acknowledge the challenges of prognostication at the individual level.^36^ Our estimates used the currently available data on the risk of death from COVID-19 across different conditions, and this multiplier should be revised as more data become available.

### Implications for policy, data and future research

A population-level understanding of the palliative care needs of people with severe COVID-19 is essential to guide efficient health service planning, but has been missing from political rhetoric around the world, much of which has been focused on intensive care capacity. Greater support for care homes through closer collaboration with primary care, specialist palliative care services and community geriatricians is needed for now and for future pandemics.^37^ Investment in palliative care training and education for social care staff so that they have the skills, competence and confidence to provide care to those approaching the end of life. Given the steep rise in deaths that have occurred in challenging circumstances, ensuring provision of bereavement support should be a priority.

COVID-19 is now the focus of much research, but very few studies are focused on palliative and end of life care.^38,39^ Data and research are urgently required to answer the following questions:

What are the best integrated models of palliative care in care homes that can be adapted for use during the COVID-19 pandemic?
What is the impact of COVID-19 on the hospice sector and how might hospice services best use their available resources?
What is the impact of COVID-19 on complex grief, and what resources are needed to support those experiencing this?
What is the impact of COVID-19 on carers supporting people with palliative care needs in community settings?

## Conclusion

Our study has highlighted the high level of need for palliative care during the COVID-19 pandemic. Healthcare systems responding to COVID-19 must consider the need for palliative care surge capacity, including the workforce and equipment, to enable people to be cared for appropriately in all settings. The need for integrated models of palliative care in care home settings is imperative. The rapid changes to practice during the pandemic provide important opportunities for research, evaluation and learning, and it is essential that new models are underpinned by evidence.

## Supplemental Material

Supplementary_material_R1 – Supplemental material for Changing patterns of mortality during the COVID-19 pandemic: Population-based modelling to understand palliative care implications Click here for additional data file. Supplemental material, Supplementary_material_R1 for Changing patterns of mortality during the COVID-19 pandemic: Population-based modelling to understand palliative care implications by Anna E Bone, Anne M Finucane, Javiera Leniz, Irene J Higginson and Katherine E Sleeman in Palliative Medicine
