# Supplementary material for: Changing patterns of mortality during the COVID-19 pandemic: Population-based modelling to understand palliative care implications
Source: Palliat Med. 2020 Jul 24;34(9):1193–201. doi: 10.1177/0269216320944810 (PMC7385436; doi:10.1177/0269216320944810)

**Supplementary material File 1**

**Table S1. Age specific Infection fatality ratio from Verity et al 2020 and adapted by Ferguson et al., 2020**

| **Age (years)** | **Infection fatality ratio, %** |
| --- | --- |
| 0 to 10 | 0.002 |
| 10 to 20 | 0.006 |
| 20 to 30 | 0.03 |
| 30 to 40 | 0.08 |
| 40 to 50 | 0.15 |
| 50 to 60 | 0.6 |
| 60 to 70 | 2.2 |
| 70 to 80 | 5.1 |
| 80+ | 9.3 |

**Table S2. Pooled adjusted effect sizes for association between selected diseases and death from COVID-19 infection (Parohan et al., 2020)**

| **Studies** |  | **Effect size (95% Confidence Interval)** | **Pooled effect size (95% Confidence Interval)** |
| --- | --- | --- | --- |
| **Cardiovascular disease (yes vs no)** | | | **3.72 (1.77, 7.83)** |
|  | Caramelo F et al. 2020 | 12.83 (10.32, 15.94) |  |
|  | Chen R et al. 2020 | 4.28 (1.14, 16.10) |  |
|  | Colombi D et al. 2020 | 3.70 (1.89, 7.25) |  |
|  | Du RH et al. 2020 | 2.46 (0.75, 8.05) |  |
|  | Liu Y et al. 2020 | 6.46 (2.33, 17.90) |  |
|  | Shi S et al. 2020 | 1.51 (0.70, 3.28) |  |
|  | Su VW et al. 2020 | 5.10 (1.68, 15.45) |  |
|  | Wang L et al. 2020 | 1.86 (1.06, 3.26) |  |
|  | Zhou F et al. 2020 | 2.14 (0.26, 17.70) |  |
| **Chronic obstructive pulmonary disease (yes, no)** | | | **3.53 (1.79-6.96)** |
|  | Caramelo F et al. 2020 | 7.79 (5.68, 10.69) |  |
|  | Guan WJ et al. 2020 | 2.68 (1.42, 5.05) |  |
|  | Liu T et al. 2020 | 4.14 (0.94, 18.23) |  |
|  | Shi S et al. 2020 | 0.37 (0.04, 3.46) |  |
|  | Su VW et al. 2020 | 7.40 (0.81, 67.72) |  |
|  | Wang L et al. 2020 | 2.24 (1.11, 4.51) |  |
|  | Zhou F et al. 2020 | 5.40 (0.96, 30.39) |  |
| **Cancer (yes, no)** | |  | **3.04 (1.8, 5.14)** |
|  | Caramelo F et al. 2020 | 6.88 (3.57, 13.26) |  |
|  | Colombi D et al. 2020 | 3.50 (1.60, 7.68) |  |
|  | Guan WJ et al. 2020 | 3.50 (1.60, 7.65) |  |
|  | Liu Y et al. 2020 | 1.89 (0.38, 9.45) |  |
|  | Shi S et al. 2020 | 1.75 (0.43, 7.14) |  |
|  | Su VW et al. 2020 | 2.70 (0.22, 33.50) |  |
|  | Wang L et al. 2020 | 0.98 (0.31, 3.11) |  |
|  |  |  |  |

**Figure S1.** **COVID-19 deaths between March 7th and May 15th 2020 in England and Wales**


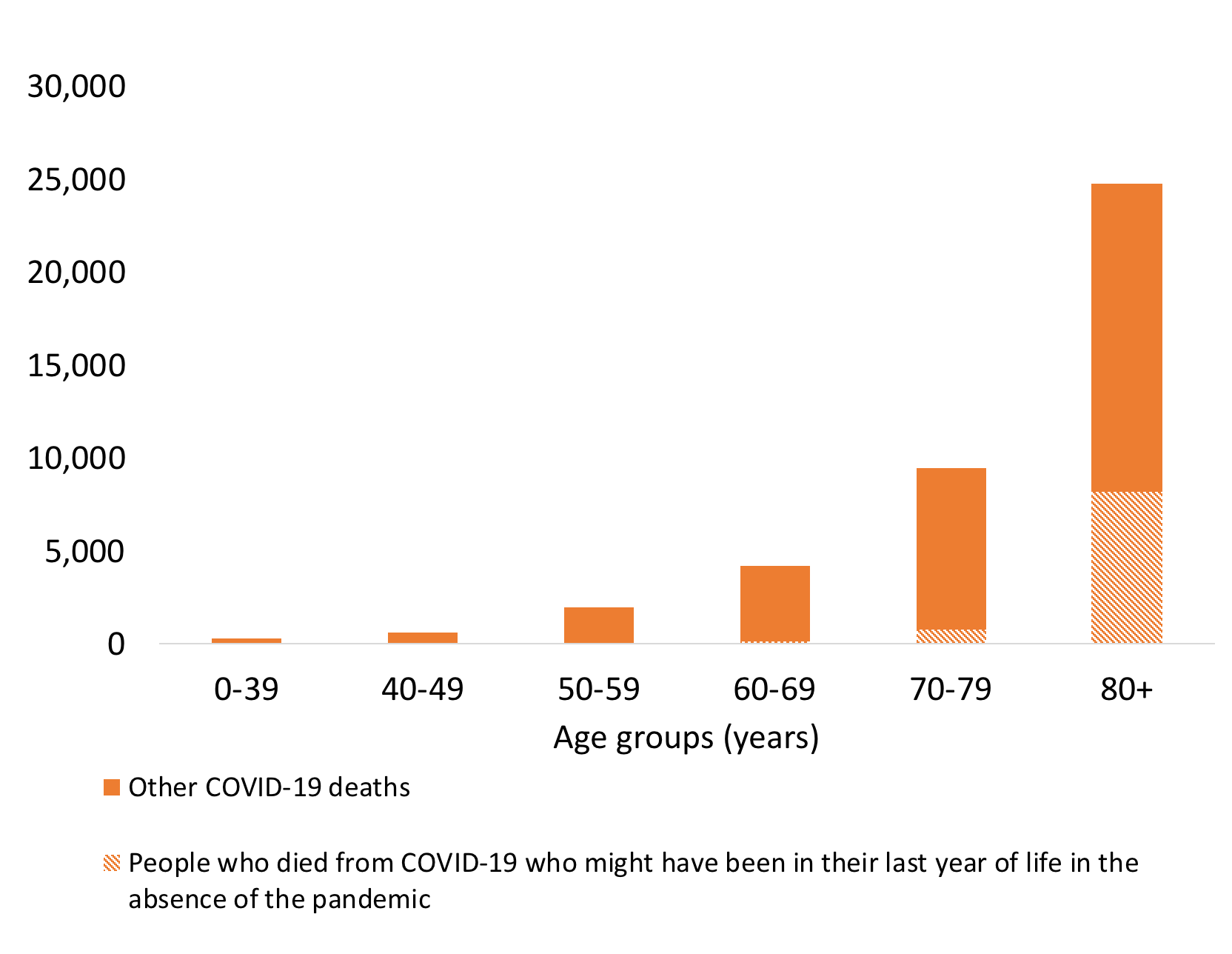

Supplement: Supplementary_material_R1 – Supplemental material for Changing patterns of mortality during the COVID-19 pandemic: Population-based modelling to understand palliative care implications [file Supplementary_material_R1.docx]
